# Targeted therapies induced depigmentation: a review

**DOI:** 10.3389/fimmu.2025.1625738

**Published:** 2025-08-08

**Authors:** Zhaoyang Wang, Meng Wang, Tianyu Wang, Xiaoxiao Yan, Zhenhua Yue, Yonghu Sun

**Affiliations:** ^1^ Dermatology Hospital of Shandong First Medical University, Jinan, China; ^2^ Shandong Provincial Institute of Dermatology and Venereology, Shandong Academy of Medical Sciences, Jinan, China

**Keywords:** targeted drugs, depigmentation, melanocytes, vaccine, autoimmunity, adverse drug reactions

## Abstract

Skin depigmentation or vitiligo-like depigmentation (VLD) is one of the most prevalent cutaneous adverse events during targeted therapies for cancers or autoimmune diseases. The depigmentation is usually with high mental burden and affect the disease treatment, some of which are even clinical markers for good prognosis. This study aimed to explore the underlying immunopathologic mechanisms of VLD induced by targeted therapy for cancer and autoimmune disease as well as vaccine, such as immune checkpoint inhibitors (e.g., programmed death 1/programmed death–ligand 1 and cytotoxic T-lymphocyte antigen-4 inhibitors), v-raf murine sarcoma viral oncogene homolog inhibitors, tyrosine kinase inhibitors, and other targeted agents. Additionally, it examined the clinical presentations, prognostic implications, and management strategies for VLD across oncologic and nononcologic contexts, including cases associated with vaccines and biologics. The development of VLD often correlates with improved therapeutic outcomes, but it presents unique challenges in balancing antitumor efficacy with patients’ quality of life. This review integrated insights from oncology, dermatology, and immunology, and underscored the need for multidisciplinary approaches to enhance the understanding, prevention, and management of these complex cutaneous adverse events.

## 1 Introduction

Targeted therapies have transformed the treatment landscape for malignancies and autoimmune diseases by specifically disrupting molecular pathways or modulating immune responses. These advancements have significantly improved therapeutic efficacy and patient survival. However, with the broader adoption of these therapies, the incidence of immune-mediated adverse events, particularly vitiligo-like depigmentation (VLD), has garnered increasing attention. VLD, often observed in patients undergoing treatments such as immune checkpoint inhibitors (ICIs), kinase inhibitors, and biologics, represents a unique intersection of therapeutic benefit and psychosocial challenge. While its occurrence is frequently correlated with enhanced treatment outcomes, the disfiguring nature of VLD profoundly affects patients’ mental health, potentially leading to anxiety, depression, and diminished quality of life. Notably, VLD is not confined to oncologic treatments but is also reported in non-cancer contexts, such as autoimmune disease management and vaccine-induced immune responses. This highlights its broad clinical relevance and the need to understand its underlying mechanisms. This review synthesizes current evidence on therapy-induced VLD, focusing on its immunopathological basis, clinical manifestations, and prognostic implications. By integrating insights from oncology, dermatology, and immunology, it underscores the necessity of multidisciplinary approaches to optimize management strategies that address not only therapeutic goals but also the psychological well-being of patients.

## 2 Targeted therapies in cancer

### 2.1 Programmed death protein 1/ligand 1 inhibitor

Programmed death 1 (PD-1) is a critical immune checkpoint molecule that regulates the immune system by downregulating T-cell activity. In the tumor microenvironment, T lymphocytes express high levels of PD-1, whereas its ligand, programmed death 1–ligand 1 (PD-L1), is expressed on the surface of tumor cells. The interaction between PD-1 on activated T cells and PD-L1 on tumor cells allows tumors to evade T-cell immune surveillance, directly suppressing tumor cell apoptosis (1, 2) PD-1 inhibitors block the binding of PD-1 to PD-L1, which relieves tumor-induced suppression of T lymphocytes, and prevents immune evasion by tumor cells, and achieving antitumor effects (3). PD-1/PD-L1 inhibitors help the immune system recognize and enhance the attack on tumor cells. However, melanoma cells and normal melanocytes share common antigens (MART-1/Melan A, gp100, tyrosinase-related protein 1, 2). During the immune destruction of tumor cells, the release of melanocyte antigens leads to the destruction of the immune privilege of normal melanocytes, which may cause normal melanocytes to be attacked by CD8+ cytotoxic T cells (3–10), thereby inducing VLD. Patients with melanoma receiving PD-1/PD-L1 inhibitors have demonstrated an increased incidence of VLD, ranging from 2% to 25%, which is considerably higher than the prevalence of vitiligo in the general population (estimated globally at 0.5% to 1%) (10). Furthermore, studies have indicated that the overall incidence of VLD in patients treated with pembrolizumab and nivolumab was 8.3% and 7.5%, respectively (5). The occurrence of VLD may indicate a better therapeutic response (3, 11, 12), and is associated with increased progression-free survival and overall survival (6, 9, 13–16). Studies have shown that patients experiencing VLD have a twofold and fourfold reduction in the risks of disease progression and mortality, respectively (17, 18). Unlike classic vitiligo, VLD induced by immune checkpoint inhibitors (ICIs) typically occurs in sun-exposed areas and presents as asymmetric, spot-like patches that gradually evolve into larger lesions (4, 10, 13, 19–22). This condition does not accompany the Koebner phenomenon and usually emerges several months after the initiation of treatment and persists even after stopping the therapy (3). Patients generally lack a personal or family history of other autoimmune diseases. Patients with VLD serum levels of CXCL10, CXCR3 expression in skin CD8 T cells, and levels of interferon-γ (IFN-γ) and tumor necrosis factor-α (TNF-α) are significantly elevated (23, 24). Elevated levels of these markers have also been found in patients with vitiligo, and the IFN-γ-CXCL9/10-CXCR3 axis is believed to play a central role in the progression and maintenance of the disease. This suggests that similar mechanisms of Th1/TC1-driven immune responses may exist in both (10). It has also been suggested that PD-1/PD-L1 function is impaired in patients with vitiligo, leading to loss of peripheral tolerance and ineffective suppression of autoreactive T cells, which in turn attack melanocytes. In the Pmel-1 vitiligo mouse model, PD-L1 fusion protein treatment can restore some pigment loss, increase the number of regulatory T cells in the skin, and reduce melanocyte-reactive T cells, with no significant side effects observed (25).

Systemic immunosuppressive therapy is the first line of treatment for vitiligo. However, this therapy is not recommended for vitiligo induced by ICIs, as it may reduce the therapeutic response to ICIs and has limited efficacy in managing this condition (4, 10). Case reports have documented that narrowband ultraviolet B (NB-UVB) phototherapy improved VLD by 15%-40% (26). Partial spontaneous repigmentation in VLD areas is also observed; however, computed tomography (CT) scans revealed disease progression (27). Repigmentation of VLD may serve as an alternative marker of disease progression or recurrence, particularly in cases responding to PD-1 therapy.

A few recent studies have reported vitiligo-like lesions in patients with other solid tumors, such as renal cell carcinoma (28, 29), non-small-cell lung cancer (NSCLC) (1, 20, 30), esophageal squamous cell carcinoma (31), hepatocellular carcinoma (32), gastric cancer (20), rectal cancer (33), and urothelial carcinoma (34), although these occurrences are relatively rare. For instance, in a case involving a patient with metastatic non-small cell lung cancer (NSCLC), vitiligo-like lesions appeared in sun-exposed areas (face, neck, forearms, and hands) after 15 months of nivolumab treatment. Immunohistochemical analysis revealed substantial CD8+ T-cell infiltration within and around the lesions (35). Antigens released from melanocyte destruction due to intense sun exposure may have caused these vitiligo-like lesions. Alternatively, the patient’s NSCLC tumor may have shared antigens with melanocytes. In another case, an elderly patient with stage III b rectal cancer developed vitiligo after treatment with toripalimab. Following radiotherapy (RT), the vitiligo lesions rapidly expanded, accompanied by severe pruritus (33). Upon discontinuation of anti-PD-1 therapy, both the vitiligo lesions and pruritus improved rapidly. Additionally, the patient was treated with topical halometasone cream and a film-forming agent containing salicylic acid, clobetasol propionate, and anthralin, which led to significant repigmentation within 1 month. Both anti-PD-1 therapy and RT possess immune-stimulating capacities, which may exhibit synergistic effects when used in combination.

### 2.2 Cytotoxic T-lymphocyte-associated antigen-4 inhibitors

Cytotoxic T-lymphocyte antigen-4 (CTLA-4) is a key regulatory factor in maintaining T-cell homeostasis and tolerance, which blocks the binding of CD28 to CD80 and CD86 (also known as B7–1 and B7-2), thereby reducing T-cell receptor signaling (3, 36, 37). Anti-CTLA-4 antibodies can reactivate and relieve the immunosuppression of CD4^+^ and CD8^+^ T cells, enhance the activation and proliferation of effector T cells, and reduce the immunosuppressive effect of regulatory T cells (Tregs) through antibody-dependent cellular cytotoxicity (ADCC), further reducing the presence of Tregs in tumor tissues (3). In the absence of CTLA−4 regulation, CD4^+^ T cells provide key assisting signals to promote CD8^+^ T cells to recognize melanocyte differentiation antigens (such as MART-1, TYR, and gp100) and migrate to the epidermis (38, 39). Activated CD8^+^ T cells release IFN-γ and TNF-α, inducing local chemotaxis mediated by the CXCL10-CXCR3 axis, causing apoptosis and clearance of melanocytes, manifested as vitiligo-like depigmentation (23, 39). Among patients with melanoma treated with anti-CTLA-4 therapy, the incidence of VLD in those receiving anti-CTLA-4 therapy ranges from 2% to 9% (3). Ipilimumab and tremelimumab are fully human monoclonal antibodies of the immunoglobulin G 1 and 2 (IgG1 and IgG2) subclasses, respectively. They block the interaction between the inhibitory molecule CTLA-4 on T cells and the B7 receptors on antigen-presenting cells. Studies have shown that 4%–11% of patients with melanoma treated with ipilimumab experience VLD (36). When combined with PD-1 inhibitors, the overall incidence of VLD rises to approximately 8% (20). Compared with nivolumab monotherapy, combination therapy with nivolumab and ipilimumab results in a shorter onset time for VLD (3.2 vs. 10.3 months). The characteristics of VLD induced by CTLA-4 inhibitors and PD-1/PD-L1 inhibitors are similar, with rare cases of near-total body depigmentation reported (40).

### 2.3 V-raf murine sarcoma viral oncogene homolog inhibitors

The v-raf murine sarcoma viral oncogene homolog (BRAF) is the most common serine-threonine protein kinase. It plays a key role in regulating intracellular signal transduction from RAS to the MEK pathway. BRAF mutations have been identified in multiple malignancies, with the BRAF V600E mutation being the most common mutation type across several cancers. In China, approximately 23.7% of patients with melanoma harbor BRAF mutations (41). Vemurafenib is the first oral drug approved for treating metastatic melanoma with BRAF V600E mutations and a significant treatment for advanced melanoma. Vitiligo-like lesions are rarely reported with BRAF inhibitors like vemurafenib or dabrafenib. Only isolated case reports, sometimes in combination with MEK inhibitors, have described depigmentation. BRAF inhibitors can enhance the expression of major histocompatibility complex (MHC) class I and class II molecules and, melanocyte differentiation antigen (MDA), while reducing PD-L1 and suppressor cells (Treg/Myeloid-Derived Suppressor Cells), reshaping the tumor microenvironment and promoting CD8^+^ T cell infiltration and activation. These CD8^+^ T cells cross-recognize melanocyte antigens (such as MART-1, gp100), combine IFN-γ/TNF-α and CXCL10–CXCR3 chemotaxis, and mediate apoptosis and clearance of normal melanocytes (21, 42). VLD typically appears in symmetric facial regions, forearms, and lower limbs. BRAF inhibitors may induce VLD by altering melanocyte function or triggering an immune response, which is often associated with favorable prognoses (43–45). Evidence suggests that four patients who developed VLD while receiving combined BRAF and MEK inhibitor therapy survived for up to 57 months (21). In another study, a patient who developed VLD following sequential treatment with nivolumab and vemurafenib showed no progression 18 months after the onset of brain metastasis, which is a common occurrence in advanced melanoma with a poor prognosis and median overall survival of approximately 4 months (46).

### 2.4 Cyclin-dependent kinase 4/6 inhibitors

Cyclin-dependent kinases (CDKs) are critical regulators of cell cycle progression, interacting with cyclin D to promote the hyperphosphorylation of retinoblastoma protein (Rb), thereby advancing the cell cycle from the G1 phase to the S phase. CDK4/6 inhibitors obstruct kinase activity, thereby blocking this G1-to-S phase transition and preventing cancer cell progression. Palbociclib, ribociclib, and abemaciclib are CDK inhibitors approved as targeted therapies for hormone receptor (HR)-positive and human epidermal growth factor receptor 2 (HER2)-negative breast cancer, the largest subtype of this malignancy. Palbociclib was the first CDK4/6 inhibitor approved by the United States Food and Drug Administration (US FDA) in 2015; it targets HR-positive/HER2-negative breast cancer. Ribociclib followed as the second CDK4/6 inhibitor approved in 2017, and abemaciclib was the third FDA-approved CDK4/6 inhibitor, indicated for use with aromatase inhibitors or fulvestrant in treating advanced or metastatic HR-positive/HER2-negative breast cancer (47). A multicenter retrospective study involving 16 patients with stage IV breast cancer reported that VLD was associated with CDK4/6 inhibitor treatment (14 of 16 were treated with ribociclib, and two of 16 with palbociclib) (48). The lesions initially appeared as small hypopigmented spots on sun-exposed areas (face, hands, and chest), gradually expanding bilaterally over the trunk and limbs. All patients reported itching. The patients achieved partial relief after monotherapy or combination treatment using topical calcineurin inhibitors and corticosteroids. Similarly, another multicenter study including 10 patients found that sun-exposed areas, such as arms and face, were the most affected. Although various therapies, such as topicals, lasers, and phototherapy, were attempted, only minimal success was observed, with mild repigmentation noted in one patient treated with ruxolitinib cream (49). Some patients experienced spontaneous depigmentation reduction within months after discontinuing the CDK4/6 inhibitors (50). A pre-inflammatory phase appears to be another characteristic of CDK4/6 inhibitor-induced VLD. In one study, a 50-year-old woman with metastatic breast cancer developed erythema and periorbital whitening after 1.5 years of ribociclib treatment, which was diagnosed as photosensitive dermatitis and vitiligo (51). Her facial rash resolved completely with twice-daily topical steroids, after which VLD appeared. This was further corroborated in a study of 16 patients, where 11 experienced a pre-vitiligo inflammatory phase characterized by a diffuse papular rash with pruritus (48). The underlying mechanism is likely multifactorial, involving both direct cytotoxic effects on melanocytes and indirect modulation of immune responses.

CDK4/6 inhibitors exert cytostatic effects by inducing G1 cell cycle arrest not only in tumor cells but also in normal proliferative cells, including keratinocytes and melanocytes. Keratinocytes play a vital role in supporting melanocyte survival and function through paracrine factors such as stem cell factor (SCF) and endothelin-1 (ET-1) (52, 53). Inhibition of keratinocyte proliferation may disrupt this supportive niche, rendering melanocytes more vulnerable to stress-induced apoptosis.

Concomitantly, the immunological landscape is significantly altered. CDK4/6 inhibitors have been shown to downregulate regulatory T cell populations and enhance MHC-I expression on both tumor and stromal cells, thereby promoting antigen presentation (54, 55). In the context of melanocyte apoptosis and subsequent release of melanocyte-associated antigens, these immune changes may foster cytotoxic T lymphocyte –mediated responses against melanocytes. Histopathologic findings from VLD cases associated with CDK4/6 inhibitors often reveal loss of melanocytes in the basal layer, CD8^+^ T cell infiltration, supporting an immune-mediated melanocytotoxic process (56). Collectively, these observations suggest that CDK4/6 inhibitors can lead to melanocyte destruction through mechanisms such as cell cycle disruption, inhibition of keratinocyte-melanocyte crosstalk, and T cell-mediated cytotoxicity. Whether the occurrence of VLD is beneficial to survival is still unclear (48, 49).

### 2.5 BCR-ABL tyrosine kinase inhibitors

Chronic myeloid leukemia (CML) constitutes 15% of adult leukemias and is characterized by the malignant proliferation of hematopoietic stem cells within the bone marrow (57). The BCR-ABL1 fusion gene encodes the BCR-ABL1 protein, which exhibits potent tyrosine kinase activity, that leads to abnormal signal pathway activation, rapid tumor cell proliferation, and inhibition of apoptosis. Targeted therapy has significantly advanced CML treatment, with BCR-ABL tyrosine kinase inhibitors (TKIs) now the most preferred treatment, which improves the 10-year survival rate from 20% to 80%–90%. TKIs include the first-generation agent imatinib, second-generation agents dasatinib, nilotinib, and bosutinib, and third-generation agents ponatinib and olverembatinib.

Although TKIs have extended the lifespan of patients with CML, they can cause adverse effects. One study reported pigmentary side effects of imatinib, with 40.9% and 3.6% of 118 patients experiencing hypopigmentation and hyperpigmentation (58). In one study, a pediatric patient with Ph-positive acute lymphoblastic leukemia (ALL) relapsed post-hematopoietic stem cell transplantation and received dasatinib. After 4 weeks of treatment, depigmented patches appeared on the neck and dorsum of the hands, and complete depigmentation of hair, eyelashes, and eyebrows was observed (59). In another study, a patient in the chronic phase of CML developed widespread depigmented macules after achieving a deep molecular response with imatinib for 1 year (60). In addition to BCR-ABL, TKIs also target multiple tyrosine kinases, such as the c-Kit proto-oncogene. c-Kit and its ligand stem cell factor (SCF) play important roles in melanogenesis, melanocyte homeostasis, and UV-induced pigmentation. Therefore, inhibition of the c-Kit/SCF signaling pathway is considered to be the cause of pigmentary side effects in patients receiving TKI treatment. A clinical example of this signaling pathway is seen in patients with piebaldism, an autosomal dominant disorder caused by mutations in the KIT oncogene, which results in the loss of melanocytes and the appearance of white spots (61).

### 2.6 Epidermal growth factor receptor tyrosine kinase inhibitors

Epidermal growth factor receptor (EGFR) is a key stimulator of cancer growth and is closely associated with tumorigenesis, making EGFR-TKIs a critical focus in anticancer drug development. EGFR-TKIs are classified into three generations; the first-generation agents include gefitinib and erlotinib, the second-generation agents include afatinib and dacomitinib, and the third-generation agents include osimertinib, aumolertinib, and furmonertinib. These are widely used to treat colorectal cancer, NSCLC, pancreatic cancer, and other malignancies. Given that the EGFR signaling pathway is essential for maintaining the integrity of the skin barrier, skin toxicity is a prevalent side effect of EGFR inhibitors. Current reports mainly focus on skin inflammation or acne-like rash, and rarely see long-term observations of white spots or skin pigment changes.

Gefitinib is a TKI that targets and inhibits EGFR. It was initially approved for NSCLC treatment. Its use has expanded to other solid tumors, such as breast, colorectal, and head and neck cancers. Gefitinib rarely causes vitiligo. In one reported case, a patient with metastatic squamous cell carcinoma of the parotid gland developed vitiligo 1 month after initiating gefitinib treatment (62). The patient’s depigmentation spread, leading to extensive and progressive loss of pigmentation across the arms, upper back, neck, face, left hip, and right chest. The depigmentation persisted even 3 years after stopping the medication. It is currently hypothesized that gefitinib and a similar tyrosine kinase inhibitor, dasatinib, may be caused by mutations in the proto-oncogene c-Kit and blockade of the melanocyte stem cell factor ligand and c-Kit signaling pathways.

### 2.7 Anaplastic lymphoma kinase - tyrosine kinase inhibitors

Anaplastic lymphoma kinase (ALK) is a receptor tyrosine kinase that belongs to the insulin receptor superfamily. It activates multiple intracellular signaling pathways, thereby regulating cell growth, transformation, and anti-apoptotic processes. ALK gene rearrangements, mutations, or amplifications have been identified in various tumors, activating downstream signaling pathways and enhancing cancer cell proliferation, growth, and invasion. ALK TKIs include the first-generation agent crizotinib, the second-generation agents ceritinib, alectinib, and brigatinib, and the third-generation agent lorlatinib. We previously reported a patient with NSCLC who developed new depigmented patches after 1 year of alectinib treatment, achieved rapid and unexpected repigmentation through laser therapy (63). Due to the rarity of such cases, we hypothesize that ALK inhibitors may negatively regulate melanocytes, leading to vitiligo-like depigmentation, but the specific mechanism remains to be further explored.

### 2.8 B-cell lymphoma-2 inhibitors

Venetoclax, was approved by the FDA in 2016 as the world’s first Bcl-2 inhibitor. Venetoclax has a high affinity for the BH3 binding domain of Bcl-2, It inhibits the overexpression of Bcl-2 in acute myeloid leukemia (AML) cells, promoting apoptosis and inhibiting cell proliferation. Venetoclax has been approved as a first-line treatment for chronic lymphocytic leukemia (CLL). The first reported case of venetoclax-induced vitiligo involved a 71-year-old woman treated for AML (64). Vitiligo initially appeared on the back of her hands and spread to her neck and chest. She had to temporarily discontinue venetoclax due to diarrhea and pancytopenia, and during this time, vitiligo completely resolved. However, shortly after resuming the drug, the condition reappeared. Another case involved a 77-year-old man with Rai stage II CLL who developed vitiligo on his limbs after 2 years of treatment (65). Bcl-2 is a key molecule for cell anti-apoptosis. Its inhibition may make melanocytes more sensitive to stress stimuli (such as light and oxidative stress), making them more susceptible to apoptosis or leading to cytotoxic T cell-mediated melanocyte destruction, causing pigmentation reduction.

### 2.9 Chemokine receptor 4 monoclonal antibody

CCR4 is mainly expressed on regulatory T cells (Tregs) and helper T cells type 2 (Th2). Tregs play a key role in maintaining immune tolerance and preventing autoimmune reactions. Mogamulizumab is an innovative defucosylated monoclonal antibody that targets C-C chemokine receptor 4 and can eliminate Sézary cells through antibody-dependent cell-mediated cytotoxicity (ADCC). In one study, three patients with Sézary syndrome developed vitiligo 6–8 months after starting mogamulizumab treatment, with depigmented patches on the face, hands, scalp, upper limbs, legs, and trunk. Mogamulizumab can also eliminate CCR4-expressing Tregs through ADCC. The depletion of Tregs may lead to a reduction in immunosuppression, resulting in enhanced activity of cytotoxic T cells (CTLs). When Tregs are exhausted, the activity of CTLs may increase, which may lead to an autoimmune attack on melanocytes. Notably, the development of vitiligo was associated with sustained complete remission or significant partial remission of mycosis fungoides (66). These cancer therapies and their association with VLD are summarized in **Table 1A**.

**Table 1A:** VLD associated with cancer therapies.

Drug class	Representative agents	Indications	VLD incidence	Proposed mechanism
PD-1/PD-L1 inhibitors	Pembrolizumab, Nivolumab, etc.	melanoma	Common (2%~25%) (10)	Enhanced immune response destroys melanocytes via shared antigens
		non-melanoma cancers	Very rare (30–34)	
CTLA-4 inhibitors	Ipilimumab	melanoma	Uncommon(2%~9%) (3)	
BRAF Inhibitors	Vemurafenib	melanoma	Very rare (43–45)	
CDK4/6 Inhibitors	Palbociclib, Ribociclib	breast cancer	Rare (48, 49)	Blocks the cell cycle of keratinocytes and melanocytes to reduce melanocyte support
BCR-ABL TKI	Imatinib	chronic myeloid leukemia	Common (40.9%) (58)	Inhibition of the c-Kit/SCF signaling pathway
EGFR TKI	Gefitinib	squamous cell carcinoma	Case reports only (62)	
ALK TKI	Alectinib	Non-small cell lung cancer	Case reports only (63)	Unknown
Bcl-2 Inhibitors	Venetoclax	Acute myeloid leukemia, chronic lymphocytic leukemia	Case reports only (64, 65)	Increased sensitivity to stressors such as light and oxidative stress
CCR4 Antagonists	Mogamulizumab	Sézary syndrome	Case reports only (66)	Depletion of Treg cells

## 3 Targeted therapies for autoimmune diseases

### 3.1 Anti-interleukin-17A monoclonal antibodies

Interleukin (IL)-17 inhibitors are biologic therapies approved for moderate-to-severe psoriasis and psoriatic arthritis. Secukinumab is a human IgG1 monoclonal antibody targeting IL-17A, whereas ixekizumab is a humanized IgG4 monoclonal antibody that binds to and inhibits IL-17A, thereby neutralizing both IL-17A homodimers and IL-17A/F heterodimers. IL-17A inhibitors rarely induce VLD. In one study, a patient with severe plaque psoriasis achieved complete skin clearance (PASI 100 response) after 4 weeks of ixekizumab treatment, but depigmented patches and plaques appeared on the face, particularly on the cheeks and chin, by week 12 (67). Vitiligo was confirmed by dermatological examination, including Wood’s lamp analysis, confirmed the diagnosis of vitiligo. Another patient with a 32-year history of psoriasis developed extensive new vitiligo in the trunk, limbs, and face after switching from secukinumab (discontinued due to side effects) to ixekizumab for 11 months (68).

The mechanism by which IL-17 inhibitors contribute to vitiligo remains unclear. One theory suggests that IL-17 inhibitors may induce an imbalance in the T helper cell 17 (Th17)/T helper cell 1 (Th1) response, with cytokines secreted by Th1 cells activating natural killer cells and cytotoxic CD8+ T cells targeting melanocytes. In one study, a patient’s psoriasis and VLD were both managed successfully after discontinuing IL-17A inhibitors and switching to cyclosporine. Within 3 months of cyclosporine use, 75% repigmentation was achieved (69). This effect could be attributed to cyclosporine’s broad T-cell calcineurin-inhibitory activity, which does not disrupt the Th1/Th17 balance, thus controlling both psoriasis and vitiligo.

In a patient treated with secukinumab, previously stable depigmented patches for over 2 years became larger and more pronounced (70). Skin biopsy showed an absence of epidermal melanocytes and gp100 immunoreactivity. Compared with pretreatment psoriatic lesions, posttreatment vitiligo lesions displayed higher staining levels of CD8, IFN-γ, and CXCL10, whereas staining levels of IL-17A and TNF-α were lower. The progression of vitiligo halted following topical tacrolimus therapy. Topical steroids have also shown some efficacy in some patients (71).

In some studies, vitiligo developed after treatment with adalimumab. Discontinuing adalimumab and initiating secukinumab led to gradual improvement in both vitiligo and psoriatic symptoms, with nearly complete repigmentation in depigmented areas after 1 year of treatment (72). Similarly, a 1-year-old patient with generalized pustular psoriasis developed segmental vitiligo following acitretin treatment (73). Despite a 4-month regimen of topical steroids and tacrolimus, vitiligo continued to spread to the face and trunk. After initiation of secukinumab treatment along with topical steroid use, the patient’s pustular psoriasis fully resolved, vitiligo progression stopped, and partial repigmentation was observed after three doses.

### 3.2 Anti-TNF-α monoclonal antibodies

TNF-α is a key mediator of inflammation and is targeted by several anti-TNF agents, including infliximab, adalimumab, and etanercept. To date, approximately one million patients worldwide have received anti-TNF therapy for conditions such as ankylosing spondylitis, Crohn’s disease, ulcerative colitis, rheumatoid arthritis, psoriasis, and psoriatic arthritis. Also, increased TNF-α levels have been observed in skin samples from patients with vitiligo. TNF-α appears to be a key factor in the pathogenesis of vitiligo, as it inhibits melanocyte proliferation and function.

Paradoxically, cases of new-onset vitiligo or worsening of existing vitiligo have also been reported during anti-TNF therapy. For example, new or expanded vitiligo was found in patients treated with adalimumab (74), infliximab (75), and golimumab (76), with improvements observed upon drug discontinuation or use of adjunctive therapies such as tacrolimus ointment and excimer laser therapy (80, 83, 84). A 10-year cohort study indicated that patients receiving anti-TNF therapy had approximately twice the risk of developing vitiligo compared with those receiving conventional treatment (77). Among anti-TNF drugs, patients treated with etanercept had the highest risk of developing vitiligo, followed by infliximab and adalimumab. In patients younger than 40 years, the risk of developing vitiligo was 3.7 times higher in the anti-TNF group compared with the nonexposed group.

Several theories have been proposed to explain the development of autoimmunity during anti-TNF therapy (74). *In vivo* studies have indicated an increase in nucleosomes, which are major self-antigens released during apoptosis, potentially leading to subsequent autoantibody induction (78). Additionally, cytotoxic T cells are thought to play an essential role in suppressing autoreactive B cells, and TNF blockade may weaken this suppression, possibly allowing autoimmunity to emerge (79). Moreover, TNF inhibition may trigger cytokine shifts and activate autoreactive T cells in the epidermis, destroying melanocyte cells (80).

### 3.3 Anti-IL-12/23 monoclonal antibodies

Ustekinumab is a monoclonal antibody targeting the shared p40 subunit of IL-12/23, approved for the treating of moderate-to-severe plaque psoriasis, psoriatic arthritis, and inflammatory bowel disease. In one study, a patient with a 4-year history of psoriatic arthritis developed depigmented lesions on the dorsal side of the fingers by week 16 of ustekinumab treatment, which appeared white under Wood’s lamp examination (81). Although Ustekinumab inhibits both the IL-12 and IL-23 pathways, its inhibition of the Th17 axis is more significant in real-life settings (82). In the context of Th17 inhibition, the Th1 immune response may be relatively active due to lack of regulation (83), thereby enhancing the activation of the IFN-γ/CXCL10 axis, promoting CXCR3^+^ CD8^+^ T cell recruitment and melanocyte destruction, and inducing or aggravating vitiligo. This hypothesis still needs to be confirmed by more experiments, but it provides a reasonable immunological basis for explaining Ustekinumab-related vitiligo.

### 3.4 Anti-CD52 monoclonal antibodies

Multiple sclerosis (MS) is a chronic autoimmune T-cell-mediated disease that affects the central nervous system and is primarily seen in young adults. It occurs when the immune system attacks the myelin sheath and, eventually, axons and neurons, causing inflammation and degeneration along the neural axis (84). Alemtuzumab is a humanized monoclonal antibody targeting the glycoprotein CD52, which is expressed on the surface of T and B lymphocytes, natural killer cells, monocytes, and macrophages. Initially approved for treating CLL, alemtuzumab has been used for several years to treat relapsing-remitting multiple sclerosis (RRMS) (85). The first case of nonsegmental vitiligo (NSV) was observed in 2018 in a patient who developed NSV 5 months after the initial cycle of alemtuzumab (86). Another patient with RRMS presented with halo nevi-like depigmentation across the skin, with immunohistochemistry revealing melanocyte loss in the depigmented halo areas and T-cell infiltration in the upper dermis (87). Notably, this patient showed about a sixfold increase in anti-tyrosinase antibodies and about a threefold increase in anti-tyrosinase-related protein-1 antibodies.

In another study, a patient with RRMS receiving alemtuzumab developed acquired hemophilia (AHA) alongside an expansion of stable cervical vitiligo in multiple body areas (84) Following alemtuzumab, B cells recover faster than T cells during the immune reconstitution phase. This T-cell regulation deficiency allows uncontrolled B-cell activity, potentially increasing autoimmunity in certain cell populations (88, 89). This mechanism may explain the induction of melanocyte-specific antibodies in NSV and halo nevus-like depigmentation following alemtuzumab. Ruck and colleagues reported that three cases where patients developed vitiligo approximately 1 year after treatment initiation (90). An increase in activated CD8+ T cells was observed at vitiligo onset in alemtuzumab-treated patients compared with those without vitiligo, suggesting a role for these cells play a role in the pathogenesis of depigmentation. Alemtuzumab selectively depletes circulating T cells, including regulatory T cells, while sparing skin-resident memory T cells. According to a recent study in multiple sclerosis patients, peripheral CD4^+^CD25^+^FoxP3^+^ Tregs were profoundly reduced within one week after treatment, with only partial recovery over months (91). In contrast, TRM cells in non-lymphoid tissues such as the skin express lower levels of CD52 and are largely unaffected (92). The transient loss of Treg-mediated immune regulation may allow skin TRM—particularly CD8^+^ IFN-γ–producing cells—to become activated in a pro-inflammatory microenvironment. This imbalance can promote melanocyte destruction and may underlie the development of VLD observed in patients treated with alemtuzumab. Furthermore, anti-inflammatory cytokines, including IFN-γ, TNF-α, IL-6, and IL-21, are released during alemtuzumab therapy, which may also contribute to VLD development.

### 3.5 IL -4 receptor antagonist

Dupilumab is a fully human monoclonal antibody (IgG4 type) targeting the IL-4 receptor alpha (IL-4R-α) subunit. It inhibits IL-4/IL-13 signaling pathways, demonstrating high efficacy and tolerability in atopic dermatitis (AD) management. In a patient with AD with a small patch of NSV on the forehead, dupilumab significantly improved AD symptoms, including nodules and itching (93). However, after initiating dupilumab, the vitiligo patch expanded rapidly causing gray hair. Phototherapy and topical corticosteroids proved ineffective. After discontinuing dupilumab, slight repigmentation was observed over the following 17 months, although the size of remained unchanged.

Dupilumab acts by blocking T helper cell 2 (Th2) cytokines, potentially shifting the immune balance toward Th/Tc1 polarization. In vitiligo, CD4+ and CD8+ T cells play crucial roles by producing Th1/Tc1 signature cytokines such as IFN-γ and TNF-α. The inhibition of Th2 cytokines by dupilumab may activate Th/Tc1 cells and CD8+CD49a+ tissue-resident memory T cells in small vitiligo lesions, potentially leading to the expansion of NSV-affected skin patches.

### 3.6 IL-6 receptor antagonist

Tocilizumab is a humanized monoclonal antibody targeting the IL-6 receptor. It was approved for treating rheumatoid arthritis and juvenile idiopathic arthritis (JIA). A patient with JIA developed multiple halo nevi after 3 years of tocilizumab treatment, which was unresponsive to UVB therapy (94). Another 33-year-old woman developed halo naevi, vitiligo, and diffuse alopecia areata after tocilizumab treatment. The researchers speculated that IL-6 may cause immune-mediated damage to melanocytes by affecting the balance between Tregs and Th17 cells (95). A summary of these autoimmune disease therapies that can induce VLD is provided in **Table 1B**.

**Table 1B:** VLD associated with treatments for autoimmune diseases.

Drug class	Representative agents	Indications	VLD incidence	Proposed mechanism
IL-17 Inhibitors	Secukinumab	psoriasis and psoriatic arthritis	Very rare (67, 68)	Imbalance in the T helper cell 17/T helper cell 1 response
TNF-α Inhibitors	Etanercept	Crohn’s disease, ulcerative colitis, rheumatoid arthritis, psoriasis and psoriatic arthritis	Rare (77)	Increased release of autoantigens, induction of autoantibodies; decreased suppression of autoreactive B cells by cytotoxic T cells
IL-12/23 Inhibitors	Ustekinumab	psoriasis and psoriatic arthritis	Very rare (81)	Imbalance in the T helper cell 17/T helper cell 1 response
CD52 Monoclonal Antibodies	Alemtuzumab	multiple sclerosis	Very rare (86, 87)	Depletion of regulatory T cells but preservation of skin-resident memory T cells
IL-4R Antagonists	Dupilumab	atopic dermatitis	Case reports only (93)	Blocking T helper 2 cytokines may shift the immune balance toward Th/Tc1 polarization
IL-6R Antagonists	Tocilizumab	rheumatoid arthritis and juvenile idiopathic arthritis	Case reports only (94, 95)	Affects the balance of Tregs and Th17 cells

## 4 Vaccines

Several studies have reported the onset of vitiligo-like depigmentation (VLD) following Coronavirus Disease 2019 (COVID-19) vaccination. These cases involved individuals of various ages, sexes, and ethnic backgrounds, and were associated with a range of COVID-19 vaccines, including Pfizer-BioNTech, Moderna, AstraZeneca, and Sinovac (96). VLD typically emerged within a few weeks post-vaccination, with an average onset of 2.1 weeks. In certain cases, a strong temporal relationship between vaccination and VLD development was observed (97).

Multiple immunological mechanisms have been proposed to explain this phenomenon. One possibility is molecular mimicry, in which structural similarities between viral antigens and self-antigens lead to cross-reactive immune responses. In genetically predisposed individuals, exposure to pathogens or vaccines may activate T or B cells that recognize both viral and host antigens, breaking immune tolerance and redirecting immune responses against melanocytes (98, 99). COVID-19 vaccines specifically introduce the SARS-CoV-2 spike (SP) glycoprotein to elicit immune memory and neutralizing antibodies. Notably, numerous shared heptapeptides have been identified between fragments of the SARS-CoV-2 SP and proteins within the human proteome, suggesting a molecular basis for autoimmune cross-reactivity (100). This peptide overlap may help explain immune-mediated events, including VLD, following both SARS-CoV-2 infection and vaccination.

Tissue-resident memory T cells (TRMs), which play a central role in vitiligo pathogenesis, may be aberrantly activated through molecular mimicry involving the spike protein (96). Beyond mimicry, bystander activation—whereby viral infection induces the release of sequestered self-antigens—has also been implicated (98).

SARS-CoV-2 infection itself may similarly contribute to vitiligo onset. The virus induces a hyperinflammatory state known as the “cytokine storm,” characterized by excessive cytokine release and immune cell activation, leading to systemic oxidative stress and tissue damage (101). As oxidative stress is a known contributor to melanocyte destruction in vitiligo, this may serve as a mechanistic link. Moreover, SARS-CoV-2 stimulates dendritic cells to produce high levels of interferons (IFNs), particularly type I and II, which are crucial cytokines in vitiligo pathogenesis (102).

It has also been hypothesized to involve upregulation of CD38 expression on memory spike-specific CD8+ T cells early after vaccination or infection. Elevated CD38 may lead to nicotinamide adenine dinucleotide (NAD^+^) depletion, thereby impairing the SIRT1/Nrf2 antioxidative signaling axis. This cascade may result in dysregulated MAPK activity and FAS-mediated apoptosis of melanocytes (103). Genetic and epigenetic variation in the CD38–NAD^+^–SIRT1 pathway may influence susceptibility to VLD in some individuals.

Clinically, VLD after COVID-19 vaccination often presents as symmetrically distributed depigmented patches, in contrast to the more localized or asymmetric patterns often seen with immune checkpoint inhibitor (ICI)-induced VLD (104–106). Nonetheless, localized or segmental variants have also been reported (99). Commonly affected areas include the face, neck, hands, feet, arms, and legs (101, 107). some patients, partial repigmentation, especially on the face, has been observed after two months of narrowband UVB (NB-UVB) therapy (108).

A few illustrative cases have been described. For instance, a 66-year-old man with a 10-year history of stable vitiligo on prednisone (15 mg/day) experienced a marked flare following a second dose of the mRNA vaccine (Comirnaty), with new lesions appearing on the limbs, face, perioral area, trunk, axillae, and genital region (107).

Despite these case reports, large-scale epidemiological data have not demonstrated a significant increase in vitiligo risk post-vaccination. A population-based study involving over 3.8 million vaccinated individuals and a matched cohort of unvaccinated controls found no statistically significant elevation in vitiligo incidence among the vaccinated group (109).

In conclusion, while isolated cases suggest a potential link between COVID-19 vaccination and VLD, the overall risk appears low. Further mechanistic and epidemiological studies are warranted to clarify the autoimmune implications of both SARS-CoV-2 infection and vaccination. The key details of VLD cases following vaccination are presented in **Table 1C**.

**Table 1C:** VLD following vaccination.

Drug class	Representative agents	Indications	VLD incidence	Proposed mechanism
COVID-19 Vaccines	BNT162b2	COVID-19	Very rare (109)	Molecular mimicry, bystander activation

Frequencies are based on literature reports.

“Common” ≥10%; “Uncommon” 1–10%; “Rare” <1% There are few clinical studies or case series; “Very rare” Mainly case reports, but there are more than two independent literature; “Case reports only” indicates isolated reports.

References are provided where available.

*ALK*, anaplastic lymphoma kinase; *Bcl-2*, B-cell lymphoma-2; *BRAF*, v-raf murine sarcoma viral oncogene homolog, *CDK*, cyclin-dependent kinases; *CCR4*, chemokine receptor 4; *COVID-19*, coronavirus disease 2019; *CTLA-4*, cytotoxic T-lymphocyte-associated atigen-4; *EGFR*, epidermal growth factor receptor; *IL-4R*, interleukin-4 receptor; *IL-6R*, interleukin-6 receptor; *IL-17*, interleukin-17; *IL-12/23*, interleukin-12/23; *PD-1*, programmed death 1; *PD-L1*, programmed death 1–ligand 1; *Th1/2/17*, T helper cell 1/2/17; *TNF*α, tumor necrosis factor-α.

## 5 Discussion

**Figure 1:**
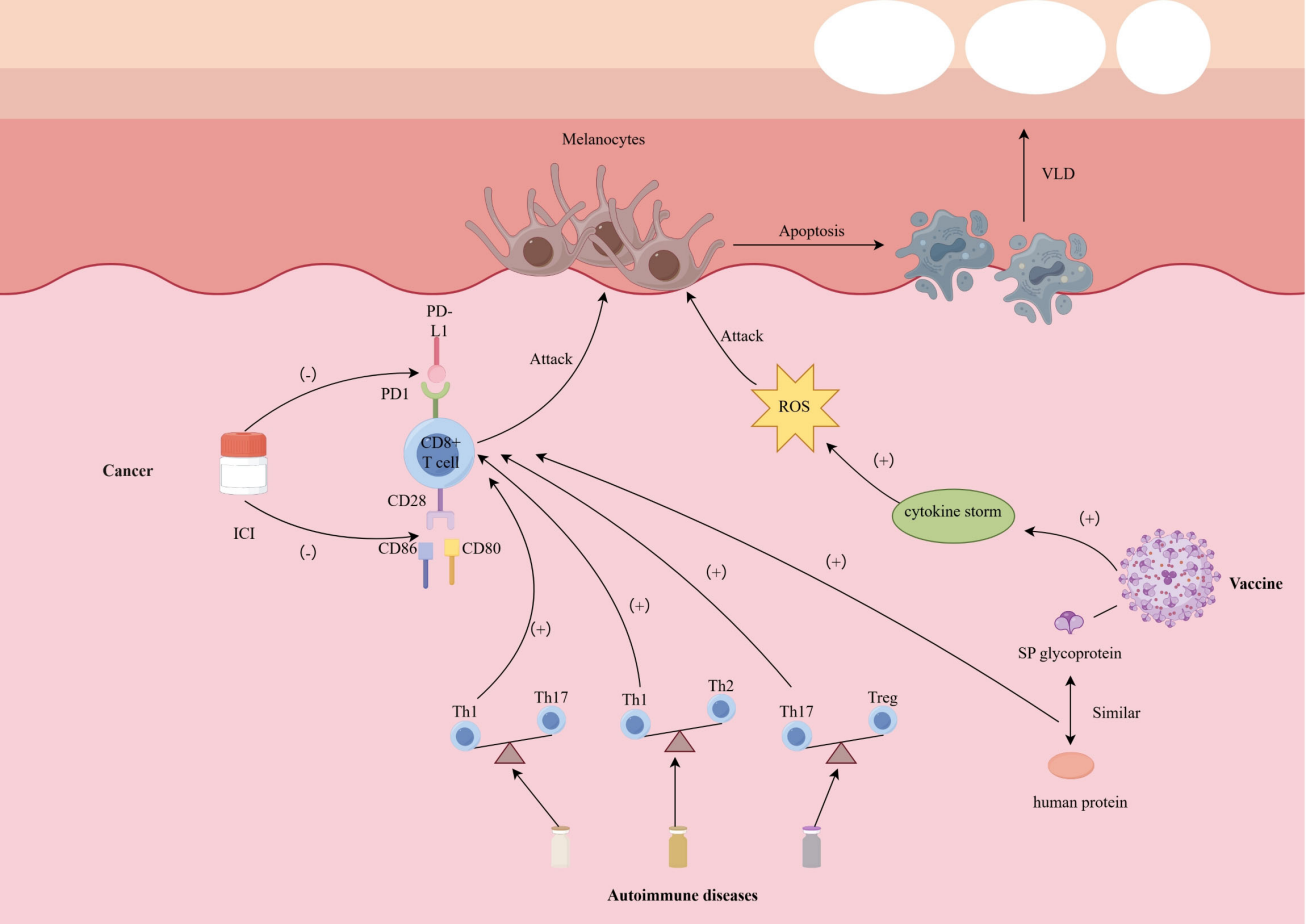
Possible mechanism of depigmentation induced by targeted therapies. *CD 28/80/86* cluster of differentiation 28/80/86, *ICI* immune checkpoint inhibitor, *PD-1* programmed death 1, *PD-L1* programmed death 1–ligand 1, *SP* spike, *ROS* reactive oxygen species, *Th1/2/17* T helper cell 1/2/17, *VLD* vitiligo-like depigmentation.

An important question should be discussed, why are melanocytes destroyed in targeted therapy (**Figure 1**)? In the autoimmune phenomenon caused by targeted therapy and immune checkpoint inhibitors, melanocytes are more easily misidentified and destroyed by the immune system than keratinocytes, hair stem cells, thyroid follicular cells or pancreatic β cells. The reason is that they have a highly immunogenic antigen spectrum, are located in immune-active tissues, and are susceptible to cytotoxic T cell responses.

First, melanocytes express a series of melanin synthesis-related antigens, such as tyrosinase, gp100, MART-1 (Melan-A), TRP-1, TRP-2, etc. These antigens are not only widely studied as immunotherapy targets in melanoma cells, but also expressed in normal melanocytes. Since ICIs treatment relieves T cell tolerance mediated by PD-1/PD-L1 or CTLA-4, CD8+ T cells that originally remained unresponsive to these “self” antigens are activated, thus triggering cross-attacks on normal melanocytes expressing the same antigens (on-target, off-tumor effect) (110). Compared with other tissues, these antigens of melanocytes are more easily recognized by T cells and induce cytotoxic immunity.

Secondly, melanocytes are located in the basal layer of the epidermis and the upper part of the hair follicle (bulge region), and are an important component of the skin, an immune-active organ. There are a large number of dendritic cells (DCs), Langerhans cells (LCs), and resident memory T cells in the skin. Targeted therapies can modulate the tumor or skin microenvironment and promote innate immune activation. For instance, kinase inhibitors or tumor-directed cytotoxic agents induce immunogenic cell death, leading to the release of damage-associated molecular patterns (DAMPs), such as HMGB1 and ATP, which APCs including dendritic cells and Langerhans cells (111). Upon activation, these APCs upregulate co-stimulatory molecules and secrete Th1-polarizing cytokines such as IL-12, TNF-α, and Type I interferons (IFN-α/β) (112). These factors contribute to a pro-inflammatory and pro-apoptotic immune milieu that enhances local T cell recruitment and activation. In the skin—a highly immune-active organ—TRM and recruited cytotoxic CD8+ T cells may recognize melanocyte-derived antigens presented via MHC-I and execute direct killing through perforin/granzyme pathways or IFN-γ-mediated apoptosis. The resultant destruction of melanocytes underlies the vitiligo-like depigmentation observed during or after targeted therapies. Although keratinocytes are widely distributed in the epidermis, they do not express these immunodominant antigens and lack sufficient co-stimulatory signals, making them less susceptible to attack by CD8+ T cells; hair follicle stem cells are also in a relatively immunosuppressive “privileged zone” and have low MHC I expression and local TGF-β/IL-10-mediated immune regulation (113).

In addition, different cell types differ in their responsiveness to IFN-γ signals. Studies have shown that melanocytes can significantly upregulate the expression of MHC class I molecules, chemokines such as CXCL9/10 under IFN-γ stimulation, further recruit CXCR3+ T cells to migrate to the skin, and strengthen the positive feedback of immune attack; this mechanism has been repeatedly confirmed in a variety of vitiligo models and ICI-related vitiligo (114). Although thyroid cells and pancreatic β cells may also undergo inflammatory destruction during ICI treatment (such as autoimmune thyroiditis with an incidence of approximately 10% and type 1 diabetes with a lower incidence of approximately 1%), this usually depends on a specific genetic susceptibility background (such as TPO antibody positivity or HLA susceptibility) and does not have broadly consistent target antigen characteristics like melanocytes (115).

VLD is a shared cutaneous phenotype observed across various clinical scenarios; however, its incidence and underlying mechanisms differ markedly among cancer, autoimmune diseases, and vaccine-related conditions. In cancer patients, particularly those receiving immune checkpoint inhibitors, VLD is relatively common (2%–25%) and is closely associated with enhanced antitumor immunity. This association is attributed to the breakdown of immune tolerance and antigenic overlap between melanoma cells and normal melanocytes, which triggers a robust CD8^+^ T cell response. In contrast, the incidence of VLD is substantially lower during treatment of autoimmune diseases with biologics. Agents such as anti–TNF-α and IL-17 inhibitors may disrupt the Th17/Th1 balance or impair regulatory T cell function, thereby inadvertently enhancing IFN-γ–mediated melanocyte destruction. Notably, both classic vitiligo and drug-induced VLD share key immunologic features, including IFN-γ/CXCL10-mediated chemotaxis and the involvement of tissue-resident memory T cells and melanocyte-specific CD8^+^ T cells. These shared mechanisms support the notion that VLD reflects an exaggerated Th1-dominant immune milieu.

Since the widespread administration of COVID-19 vaccines, vaccine-associated VLD has been increasingly reported, though it remains rare and largely confined to case reports. Proposed mechanisms include molecular mimicry and transient interferon surges; however, large-scale cohort studies have not demonstrated a statistically significant increase in vitiligo risk post-vaccination.

Compared with previous literature, the present study shares certain common findings but also provides several notable extensions. Consistent with earlier reports, our review identifies ICIs and BCR-ABL tyrosine kinase inhibitors as the most frequently implicated antitumor agents associated with VLD (116). However, we further expand the spectrum of implicated drugs to include not only tumor-targeted agents, but also small-molecule therapies increasingly used for autoimmune diseases, as well as COVID-19 vaccines, which have become globally prevalent in recent years. In addition, we provide a more comprehensive summary of the potential immunologic mechanisms linking these agents to VLD.

Currently, treatment options for VLD—particularly that induced by ICIs and other targeted cancer therapies—are limited, as systemic immunosuppression may compromise antitumor efficacy. While narrowband ultraviolet B (NB-UVB) phototherapy and topical agents have demonstrated variable success, there is no standardized management strategy. Emerging therapeutic approaches for vitiligo are under investigation and target multiple immune pathways, including IFN-γ inhibitors, CXCL10/CXCR3 antagonists, Janus kinase (JAK) inhibitors, PD-1/PD-L1 modulation, and HSP70i DNA-based therapies (117). Topical or oral JAK inhibitors, such as ruxolitinib and tofacitinib, have shown promise in classic vitiligo by suppressing the IFN-γ–STAT1–CXCL10 axis and may hold potential for the treatment of VLD. However, clinical experience remains limited, and concerns persist regarding the impact of systemic JAK inhibition on immune surveillance, especially in cancer patients.

Interestingly, recent clinical trials have demonstrated encouraging results from combining JAK inhibitors with ICIs in certain malignancies, such as non-small cell lung cancer and relapsed Hodgkin lymphoma (118, 119). They are still in the clinical trial stage, and their effects on VLD are still unknown. Carefully designed prospective studies are needed to evaluate whether localized or short-term JAK inhibition can mitigate VLD without compromising oncologic outcomes. A more nuanced understanding of VLD pathogenesis may facilitate the development of targeted interventions and inform interdisciplinary management strategies.

## 6 Conclusions

VLD significantly affects the quality of life and mental health of patients because of its disfiguring appearance. Systemic immunosuppressive therapies are commonly employed for treating vitiligo, but in the context of VLD induced by targeted therapies, such treatments may reduce antitumor efficacy. Therefore, the management of VLD often relies on phototherapy methods, such as NB-UVB therapy.

Future research should further investigate the specific immunopathological mechanisms underlying VLD, particularly the differences in presentation among patients without melanoma. Additionally, as the variety and application of targeted therapies expand, understanding how to better prevent and manage these cutaneous adverse reactions is crucial for improving treatment outcomes. Future studies should delve deeper into the molecular mechanisms of targeted therapies, collect clinical data, and foster interdisciplinary collaboration to enhance the understanding of these complex cutaneous adverse events. This approach will help optimize treatment strategies to improve the quality of life of patients.
